# Increasing prevalence and local transmission of non-B HIV-1 subtypes in the French Antilles and French Guiana between 1995 and 2018

**DOI:** 10.1093/ve/veaa081

**Published:** 2020-11-27

**Authors:** Gonzalo Bello, Edson Delatorre, Vincent Lacoste, Edith Darcissac, Cécile Herrmann-Storck, Benoit Tressières, Ornella Cabras, Isabelle Lamaury, André Cabié, Benoit Visseaux, Marie-Laure Chaix, Diane Descamps, Raymond Césaire, Mathieu Nacher, Georges Dos Santos

**Affiliations:** Laboratório de AIDS e Imunologia Molecular, Instituto Oswaldo Cruz, FIOCRUZ, Rio de Janeiro, Brazil; Departamento de Biologia, Centro de Ciências Exatas, Naturais e da Saúde, Universidade Federal do Espírito Santo, Alegre, Brazil; Laboratoire des Interactions Virus-Hôtes, Institut Pasteur de la Guyane, Cayenne, Guyane Française; Laboratoire des Interactions Virus-Hôtes, Institut Pasteur de la Guyane, Cayenne, Guyane Française; Laboratory of Microbiology, University Hospital, Pointe-à-Pitre, Guadeloupe; INSERM Centre d'Investigation Clinique 1424, Centre Hospitalier Universitaire de Pointe-à-Pitre, Pointe-à-Pitre, Guadeloupe; Service de Maladies Infectieuses et Tropicales, Martinique University Hospital, Université des Antilles, Fort-de-France EA 7524, Martinique; Department of Infectious and Tropical Diseases, Dermatology, Internal Medicine, University Hospital Guadeloupe, Pointe-à-Pitre, Guadeloupe; Service de Maladies Infectieuses et Tropicales, Martinique University Hospital, Université des Antilles, Fort-de-France EA 7524, Martinique; Université de Paris, INSERM UMR 1137 IAME, Laboratoire de Virologie, AP-HP, Hôpital Bichat-Claude Bernard, Paris, France; Université de Paris, INSERM U944, Laboratoire de Virologie, AP-HP, Hôpital Saint-Louis, Paris, France; Université de Paris, INSERM UMR 1137 IAME, Laboratoire de Virologie, AP-HP, Hôpital Bichat-Claude Bernard, Paris, France; Service de Virologie, Martinique University Hospital, Université des Antilles, Fort-de-France EA 7524, Martinique; Coordination Régionale de la lutte contre le VIH (COREVIH) and Centre d'Investigation Clinique - CIC INSERM 1424, Centre Hospitalier de Cayenne “Andrée Rosemon”, Cayenne, Guyane Française; Service de Virologie, Martinique University Hospital, Université des Antilles, Fort-de-France EA 7524, Martinique

**Keywords:** HIV-1, non-B subtype, HIV cluster, Phylodynamics, French Guiana, Guadeloupe, Martinique

## Abstract

The Caribbean and South American French Overseas Territories (CSAFOT) are the regions most heavily affected by the Human Immunodeficiency Virus type 1 (HIV-1) epidemic in France. Although dominated by HIV-1 subtype B, the detection of non-B subtypes and the great proportion of HIV-positive persons born abroad demonstrated the potential for local spread of non-B subtype strains in CSAFOT. To reconstruct the epidemiologic dynamics of major non-B subtype clusters spreading in CSAFOT, we conducted phylogenetic and evolutionary analyses of 2,523 HIV-1 *pol* sequences collected from patients living in Martinique, Guadeloupe, and French Guiana from 1995 to 2018. A large variety of HIV-1 non-B subtype strains (eight subtypes, twelve CRFs, and multiple URFs) have been introduced in CSAFOT and their prevalence significantly increases over time in Martinique and Guadeloupe. We identified twelve major transmission networks of non-B subtypes (CRF02_AG and subtypes A3, C, D, and F1) that probably arose in Guadeloupe, Martinique, French Guiana, and mainland France between the late 1970s and the middle 2000s. Phylogeographic analyses support frequent non-B subtype viral transmissions within CSAFOT as well as transatlantic transmission between CSAFOT and mainland France. Domestic transmission networks of non-B subtype variants in CSAFOT comprise both men having sex with men and heterosexual individuals from different age groups. Different HIV-1 non-B subtype variants were sequentially introduced in CSAFOT between the late 1970s and the middle 2000s and are currently spreading through domestic, regional, and/or transatlantic networks of individuals from different age and risk groups.

## 1. Introduction 

The Caribbean and South American French Overseas Territories (CSAFOT), Guadeloupe and Martinique (Caribbean Islands), French Guiana (northern coast of South America), are the regions of France the most heavily affected by the Human Immunodeficiency Virus type 1 (HIV-1) epidemic. In 2015, the rate per million inhabitants of new HIV diagnoses in Martinique (214), Guadeloupe (342), and French Guiana (743) was well above the mean rate in France (89) (Six and Quet 2016). Although in CSAFOT, HIV has primarily spread through heterosexual contact and the epidemic is often considered to be ‘generalized’ (particularly in French Guiana where prevalence in pregnant women exceeds 1%) (Cabié, Georger-Sow and Nacher 2005; Pan American Health Organization 2012), HIV reaches a high prevalence in some vulnerable groups such as migrants, crack cocaine users, and/or men who have sex with men (MSM) (Nacher et al. 2010; Klingelschmidt et al. 2017; Parriault et al. 2017).

HIV-1 subtype B is the predominant lineage in mainland France, but the prevalence of non-B subtypes is increasing (Chaix et al. 2013; Visseaux et al. 2020). This changing molecular epidemiologic pattern was attributed to both increased migration of HIV-infected individuals from non-European countries and to local transmission of non-B subtypes, particularly among MSM (Brand et al. 2014, 2017; Chaillon et al. 2017). While the HIV-1 epidemic in CSAFOT seems to be also mostly driven by subtype B, the previous identification of non-B subtypes in Martinique and French Guiana (Desgranges et al. 1996; Ouka et al. 1998; Kazanji et al. 2001; Darcissac et al. 2016) and the great proportion of HIV-positive persons born abroad among those living in CSAFOT (up to 80%, French Guiana) (Cabié, Georger-Sow and Nacher 2005; Nacher et al. 2018), demonstrated the potential for introduction and local dissemination of non-B subtype strains in these French American regions.

The aim of the present study was to explore the origin and dissemination dynamics of HIV-1 non-B subtype variants circulating in CSAFOT. To this end, we analyzed 2,543 HIV-1 *pol* sequences collected from patients living in CSAFOT from 1995 to 2018. Socio-demographic, geographic, and viral sequence data were combined with phylogenetic and molecular clock methods to identify non-B transmission networks and reconstruct their spatiotemporal dynamics.

## 2. Materials and Methods

### 2.1. Study population

HIV-1 *pol* sequences covering the complete protease and part of the reverse transcriptase regions (nucleotides 2,253–3,275 of reference strain HXB2) were obtained from adult patients accessing clinical care at the University Hospital of Martinique (Fort-de-France, Martinique) between 1995 and 2018, at the University Hospital of Guadeloupe (Pointe à Pitre, Guadeloupe) between 1999 and 2014, or at the Institut Pasteur de la Guyane (Cayenne, French Guiana) between 2006 and 2017. In the three locations all HIV-1 genotyping tests were prescribed according the same French national guidelines that didn’t recommend test prior entry to care until 2006 (Yeni 2006). Drug resistance sequences were established either by the Virology laboratory of the University Hospital of Martinique (Guadeloupe, Martinique) or by the Institut Pasteur de la Guyane (French Guiana). Only one sequence per subject was selected and if several sequences were available for a patient the first collected was selected. The epidemiologic data were extracted from eNadis/Dat’AIDS^®^, a computerized medical record.

### 2.2. Ethical statement

All phylogenetic and statistical analyses were performed on de-identified database to protect patient’s anonymity. The Dat’AIDS cohort is approved by the French ‘Commission Nationale Informatique et Liberté’ (Registration number: 2001/762876/nadiscnil.doc). The study was based on routine patient follow-up biological data and did not involve any additional sampling. Ethical approval was not needed for this study in accordance with French laws and regulations.

### 2.3. HIV-1 subtyping

HIV-1 *pol* sequences were aligned with reference sequences representative of HIV-1 group M subtypes and circulating recombinant forms (CRFs) available in the Los Alamos HIV Sequence Database (http://hiv-web.lanl.gov; accessed 15 Jul 2018) using the ClustalW program (Thompson et al. 1997). Codons associated with major antiretroviral drug resistance positions in protease (*n *=* *12) and reverse transcriptase (*n *=* *21) were excluded. Subtyping of HIV-1 sequences was first performed using the REGA HIV subtyping tool v2 (de Oliveira et al. 2005) and confirmed by bootscanning using SimPlot software v3.5.1 (Lole et al. 1999) Maximum Likelihood (ML) phylogenetic analyses using the PhyML v3 program (Guindon et al. 2010) (see Supplementary Data).

### 2.4. Identification of non-B subtype transmission clusters

HIV-1 sequences from the most prevalent clades circulating in CSAFOT were aligned with reference sequences of the same subtypes/CRFs that were isolated in mainland France, the Caribbean, Latin America, and other geographic regions where these HIV-1 variants reach a high (>5%) prevalence. All available other CRF02_AG sequences from mainland France previously described (Chaix et al. 2013; Visseaux et al. 2020) were also added as this clade is regularly increasing and present strong clustering patterns in France. Sequences were subjected to ML phylogenetic analyses as described above and highly supported (approximate likelihood-ratio test [a*LRT*] ≥ 0.85) monophyletic clusters comprising more than five sequences from CSAFOT were selected for subsequent analyses.

### 2.5. Spatiotemporal reconstructions

Major CSAFOT non-B subtype lineages and a subset of closely related HIV-1 reference sequences identified in previous ML analyses were analyzed using a Bayesian phylogeographic approach. The evolutionary rate, the age of the most recent common ancestor (T_MRCA_; years) and the spatial diffusion pattern were jointly estimated using the Bayesian Markov Chain Monte Carlo (MCMC) approach as implemented in BEAST v1.10 (Drummond and Rambaut 2007) with BEAGLE (Suchard and Rambaut 2009) to improve run-time (Supplementary Fig. S1). MCMC chains were run for 100–200 × 10^6^ generations and convergence (Effective Sample Size > 200) and uncertainty (95% Highest Probability Density [HPD] values) in parameter estimates were assessed using the TRACER v1.7 program (Rambaut et al. 2018). Maximum clade credibility (MCC) trees were summarized with TreeAnnotator v1.10.

### 2.6. Statistical analysis

Epidemiological and demographic characteristics of the cohort included in the present study were cross-tabulated with phylogenetic clusters using Fisher’s exact test or chi2. Data were analyzed with Stata© 13.0 (Statacorp, College Station, TX). To search for temporal trends, the trend-chi2 was calculated. Statistical significance was defined as P* *<* *0.05.

## 3. Results

### 3.1. Viral diversity in the French Antilles

In this study, we analyzed 1,432 HIV-1 *pol* sequences from Martinique and 1,025 HIV-1 *pol* sequences from Guadeloupe (31%) that correspond to 51% and 31% of the total number of HIV-1 positive individuals diagnosed since the beginning of the epidemic and up to the end of the sampling interval in Martinique (*n* = 2,828) and Guadeloupe (*n *=* *3,360), respectively. In each location, 87% of the HIV-1 *pol* sequences were classified as subtype B. The most common non-B subtype variants were CRF02_AG (45.1%), D (8.6%), C (6.7%), A (3.2%), and F1 (3.2%) (Table 1, Supplementary Fig. S2). Two other pure subtypes (G, H), eleven other CRFs, and numerous unique recombinants forms (URFs) were also identified (Table 1). The most common mosaic structures among URFs were B/F1 (30/68) and B/D (19/68).

**Table 1.** Distribution of non-B subtypes in Martinique and Guadeloupe (*n* = 315).

	Martinique (*n* = 185)		Guadeloupe (*n* = 130)		Total (*n* = 315)
Subtype	*n*	%	*n*	%	%
Pure subtype					
A1	3	1.6	1	0.8	1.3
A2	2	1.1	–		0.6
A3	2	1.1	2	1.5	1.3
C	11	5.9	10	7.7	6.7
D	13	7.0	14	10.8	8.6
F1	8	4.3	2	1.5	3.2
G	4	2.2	4	3.1	2.5
H	1	0.5	–		<0.5
CRFs					
CRF02_AG	97	52.4	45	34.6	45.1
CRF01_AE	6	3.2	2	1.5	2.5
Others^a^	10	5.4	10	7.7	6.4
Unassigned					
URFs	28	15.1	40	30. 8	21.6

CRF, circulating recombinant form; URF unique recombinant form; ^a^Other CRFs (number reported: Martinique - Guadeloupe): CRF06_cpx (1-0), CRF09_cpx (0-1). CRF11_cpx (3-0), CRF12_cpx (1-0), CRF13_cpx (0-1), CRF18_cpx (1-0), CRF19_cpx (0-4), CRF24_cpx (1-0), CRF25_cpx (0-3), CRF37_ cpx (3-1).

### 3.2. Increasing prevalence of HIV-1 non-B Subtypes in the French Antilles

**Figure 1.**
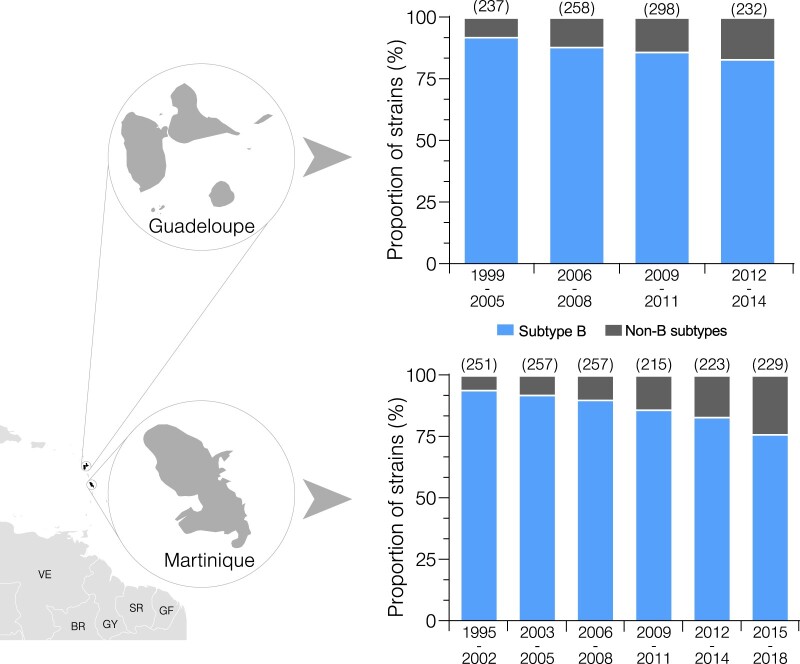
Dynamics of HIV-1 subtype B and non-B clades prevalence over time in Martinique and Guadeloupe. The subtype B/non-B subtype proportion is displayed as the overall percentage of sequences collected from Guadeloupe and Martinique per time interval. The total of sequences analyzed in each period is indicated above each column. Abbreviations: BR, Brazil; GF, French Guiana; GY, Guyana; SR, Suriname, VE, Venezuela. (Based on the map from <https://d-maps.com/> accessed 15 Oct 2019).

HIV-1 subtype B was the most prevalent variant at all time-intervals but the frequency of non-B subtype variants significantly increased over time in both Martinique (Chi2 for linear trend = 30.9, P* *<* *0.001) and Guadeloupe (Chi2 for linear trend = 3.15, P* *=* *0.07) (Fig. 1; Supplementary Table S1). The prevalence of non-B subtype-infected patients quadrupled in Martinique from 6% (1995–2002) to 24% (2015–2018) and doubled in Guadeloupe from 8% (1999–2005) to 17% (2012–2014).

### 3.3. Identification of major non-B subtype clades circulating in CSAFOT

**Figure 2.**
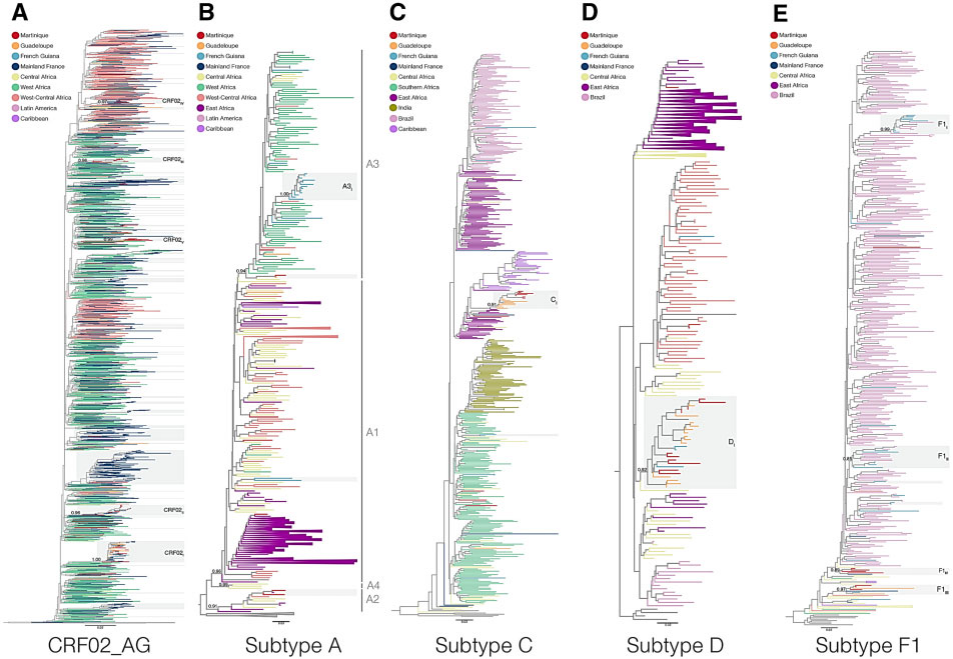
Maximum likelihood (ML) phylogenetic trees of most prevalent HIV-1 non-B clades detected in CSAFOT. Different phylogenetic trees were reconstructed for CRF02_AG (A) and subtypes A (B), C (C), D (D), and F1 (E) sequences recovered from CSAFOT and other key geographic regions. The colors of the terminal branches represent the geographic origin of each sequence as indicated in the legend of each tree. The local/regional main clusters are indicated by shaded boxes with their a*LRT* branch support values. The branch lengths are drawn to scale with the bar in the center indicating nucleotide substitutions per site.

The most prevalent HIV-1 non-B clades (CRF02_AG and subtypes A, C, D, and F1) detected in Martinique (*n *=* *136) and Guadeloupe (*n *=* *74) were aligned with sequences of the same subtype/CRF from French Guiana (*n *=* *86), mainland France (*n *=* *786), neighboring American countries and other geographic regions where these HIV-1 variants are quite frequent (>5%, Supplementary Table S2). ML phylogenetic analyses revealed multiple introductions of major non-B strains into CSAFOT (Fig. 2 and Supplementary Fig. S3). Most CSAFOT non-B subtype sequences analyzed (59%) were distributed among 12 clusters of medium/large size (*n* ≥ 5 sequences) within clade CRF02_AG and subtypes A3, C, D, and F1 (Fig. 2; Supplementary Table S3).

These clusters comprised a significant fraction of the non-B subtype sequences from Guadeloupe (66%), Martinique (63%), and French Guiana (46%), as well as a small fraction of sequences from mainland France (7%). Clusters CRF02_III_, CRF02_V_, A3_I_, C_I_, D_I_, comprised only sequences from CSAFOT and were thus defined as French American clusters. The other clusters comprise sequences from CSAFOT and mainland France (CRF02_I_, CRF02_II_, CRF02_IV_, F1_III_, and F1_IV_) or from CSAFOT and Brazil (F1_I_ and F1_II_). Clusters CRF02_III,_ A3_I_, F1_I_, and F1_II_ were detected in a single French territory, while others comprised sequences from two (CRF02_V,_ C_I_, and F1_IV_), three (CRF02_II_, CRF02_IV_, and D_I_) and four (CRF02_I_ and F1_III_) different French territories. The remaining sequences from CSAFOT were distributed in local clusters of small size (*n* < 5 sequences, 13%) or appeared as non-clustered infections (26%, Supplementary Table S4). A few CRF02_AG French Caribbean sequences (3%) branched within large French clusters mostly restricted to mainland France (Supplementary Table S4).

### 3.4. Origin and dissemination of major non-B subtype clades circulating in CSAFOT

**Figure 3.**
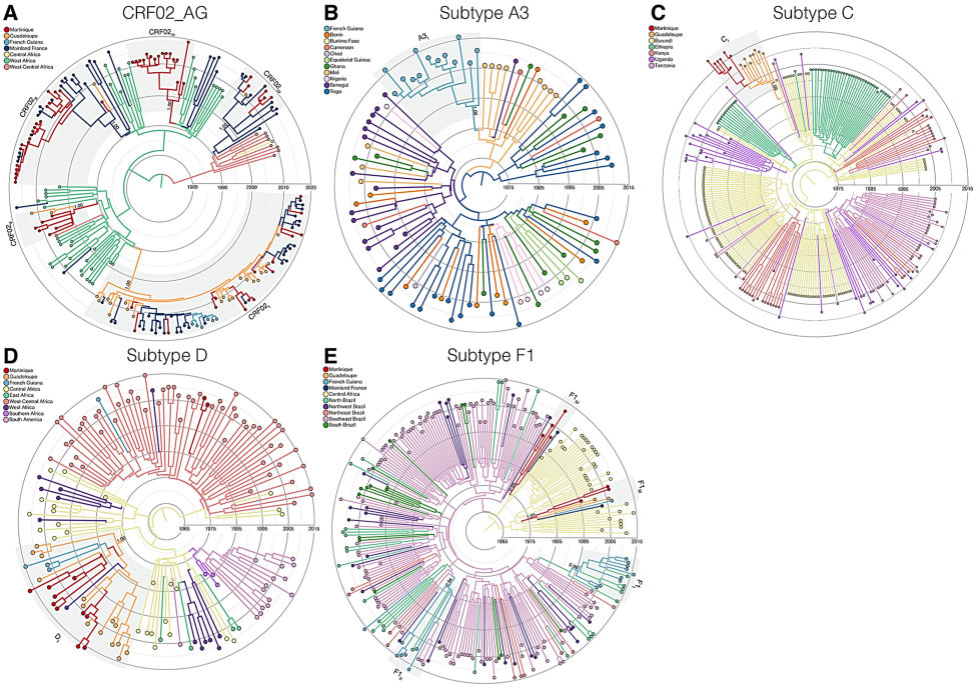
Bayesian time-scaled maximum clade credibility phylogeny of the major CSAFOT non-B subtype lineages and closely related non-CSAFOT sequences. Different molecular clock phylogenies were reconstructed for CRF02_AG (A) and subtypes A3 (B), C (C), D (D), and F1 (E). The branches’ colors represent the most probable location of their descendent nodes as indicated in each legend. The posterior (*PP*) probabilities are denoted close to key branches of each cluster (shaded boxes) while the posterior state probabilities are shown in Table 2. All branch lengths represent years, following the scale indicated by the circles.

**Figure 4.**
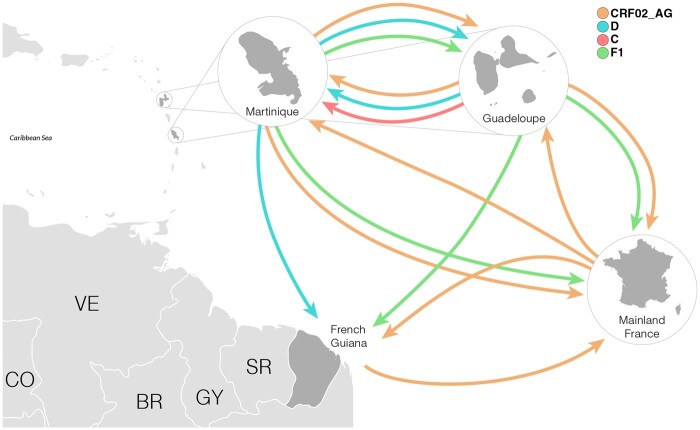
Regional migration events of non-B subtypes viral strains detected in CSAFOT. Viral migrations of non-B strains between locations (Martinique, Guadeloupe, French Guiana and mainland France) were represented by the arrows colored according to the HIV-1 clade. Abbreviations: BR, Brazil; CO, Colombia; GY, Guyana; SR, Suriname, VE, Venezuela. (Based on the maps from <https://d-maps.com/≥ accessed 15 Oct 2019).

To reconstruct the most probable source location and subsequent dispersion pattern of major non-B subtype lineages here identified in CSAFOT, HIV-1 French sequences from those clades and a subset of non-French HIV-1 reference sequences closely related in previous ML analyses (Supplementary Table S5) were analyzed using Bayesian phylogeographic reconstructions. All major non-B subtypes CSAFOT clusters displayed a high clade support in Bayesian analyses (PP > 0.93), thus confirming the ML tree topology (Supplementary Table S6). The most probable origin was traced to West Africa for clusters CRF02_I_, CRF02_II_, CRF02_III_, CRF02_V_ and A3_I_, Cameroon for cluster CRF02_IV_, Central Africa for clusters D_I_, F1_III_ and F1_IV_, Burundi for clade C_I_, and Brazil for clades F1_I_ and F1_II_ (Fig. 3 and Table 2).The clade root was most probably placed in Martinique for clusters CRF02_III_, CRF02_V_ and F1_IV_, in Guadeloupe for clusters CRF02_I_, C_I_ and D_I_, in French Guiana for cluster A3_I_, and in mainland France for clusters CRF02_II_ and CRF02_IV_ (Table 2). The root of clusters F1_I_ and F1_II_ was placed in French Guiana or Brazil and that of clade F1_III_ in Martinique or Guadeloupe with a similar probability (Table 2). The median T_MRCA_ was traced to around the late 1970s for clade D_I_, during the 1980s for clades F1_II_, F1_III_ and F1_IV_, during the 1990s for clades CRF02_II_, CRF02_III_, CRF02_V_, A3_I_ and F1_I_, and during the 2000s for clades CRF02_I_, CRF02_IV_ and C_I_ (Table 2). Our analyses support the occurrence of bidirectional disseminations of non-B strains between Martinique and Guadeloupe (CRF02_I_, CRF02_V_, C_I_, D_I_, and F1_III_), from Martinique to French Guiana (D_I_), and from Guadeloupe to French Guiana (F1_III_) as well as frequent transatlantic disseminations of non-B strains from French Caribbean islands to mainland France (CRF02_I_, CRF02_II_, F1_III_, and F1_IV_) and from mainland France to French Caribbean islands (CRF02_I_, CRF02_II_, and CRF02_IV_) and French Guiana (CRF02_I_) (Fig. 4).

**Table 2.** Origin of major non-B subtypes clusters circulating in CSAFOT.

Cluster	*n*	Origin (PSP)	Location _MRCA_ (PSP)	T_MRCA_(95% HPD)
CRF02_I_	71	West Africa (1)	Guadeloupe(0.98)	2004 (2000–2007)
CRF02_II_	34	West Africa (1)	Mainland France (0.68)	1995 (1991–1998)
CRF02_III_	16	West Africa (1)	Martinique (0.97)	1995 (1991–1999)
CRF02_IV_	13	Cameroon (1)	Mainland France (0.97)	2005 (2000–2008)
CRF02_V_	8	West Africa (0.99)	Martinique (0.55)	1996 (1992–2000)
A3_I_	13	Mali (0.97)	French Guiana (1)	1998 (1993–2003)
C_I_	16	Burundi (0.99)	Guadeloupe (1)	2003 (2001–2004)
D_I_	27	Central Africa (0.99)	Guadeloupe (0.91)	1978 (1973–1983)
F1_I_	9	Brazil (1)	Brazil-North (0.62)	1997 (1991–2002)
F1_II_	7	Brazil (0.97)	French Guiana (0.58)	1986 (1981–1991)
F1_III_	7	Central Africa (0.99)	Martinique (0.45)	1984 (1978–1989)
F1_IV_	5	Central Africa (0.99)	Martinique (0.99)	1983 (1977–1988)

### 3.5. Epidemiological characteristics of individuals belonging to major non-B subtype clades circulating in CSAFOT

The cross-tabulation of epidemiological and demographic characteristics showed significant differences between clusters and sex, mode of transmission, age, country of origin, and stage of disease (Table 3). Clusters CRF02_I_ and CRF02_II_ were mainly linked (>75%) to males with MSM-transmission; while clusters CRF02_III_, CRF02_V_, A3_I_, C_I_, D_I_, F1_III_, and F1_IV_ were mainly found (>65%) among individuals with heterosexual transmission. When looking at age, clusters CRF02_I_ and CRF02_III_ mostly (>80%) comprised young individuals (<30 years old), cluster C_I_ mostly (57%) comprised individuals >50 years old, whereas other clusters were more evenly distributed across individuals with different ages. Most individuals (≥69%) from clusters CRF02_I_ to CRF02_V_, C_I_, and F_III_ were from Martinique/Guadeloupe, cluster A3_I_ was found in French Guianese patients from the border area with Suriname as well as in individuals from Suriname, cluster D_I_ comprised a large fraction (18%) of individuals born outside of France (Congo, Dominica, and Suriname), and most individuals (10 of 12 for whom origin was known) from clusters F1_I_ and F1_II_ were from Brazil and one had a Brazilian partner. Clusters CRF02_V_ and D_I_ comprised a large proportion (>60%) of immunosuppressed individuals (<200 CD4^+^ T cells per mm^3^), clusters CRF02_I_ and CRF02_II_, F1_I_ and F1_II_ were the most frequent (>80%) in patients without advanced HIV disease (>200 CD4^+^ T cells/mm^3^). whereas remaining clusters comprised roughly similar proportions of individuals with and without advanced HIV disease. There was no significant link between clusters and viral load categorized by log10 values (P* *=* *0.148).

**Table 3.** Epidemiological characteristics of subjects in major HIV-1 non-B subtype clusters circulating in CSAFOT.

		CRF02I (*n* = 71)	CRF02II (*n* = 34)	CRF02III (*n* = 16)	CRF02IV (*n* = 13)	CRF02V (*n* = 8)	A3I (*n* = 13)	CI (*n* = 16)	DI (*n* = 27)	F1I (*n* = 9)	F1II (*n* = 7)	F1III (*n* = 7)	F1IV (*n* = 5)	P
Sex (%)	F	–	12	56	17	50	46	71	26	11	33	83	75	<0.0001
	M	100	88	44	83	50	54	29	74	89	67	17	25	
Mode of transmission (%)	MSM	87	82	6	na	–	8	–	30	na	na	–	–	<0.0001
	Hetero.	13	18	88	na	100	92	100	65	na	na	100	75	
	Others	–	–	6	na	–	–	–	4	na	na	–	25	
Age group, years (%)	≤30	85	59	81	50	33	46	–	46	56	–	60	25	<0.0001
	31–40	7	35	19	25	33	31	–	31	11	50	20	50	
	41–50	7	6	–	25	17	–	43	15	11	17	20	–	
	≥51	–	–	–	–	17	23	57	8	22	34	–	25	
CDC stage (%)	1	28	29	12	–	–	36	29	8	33	17	–	–	0.005
	2	64	53	44	–	17	18	43	31	56	67	60	50	
	3	8	18	44	–	83	45	29	61	11	17	40	50	
Country of origin (%)	MQ	28	82	69	100	100	–	86	18	–	–	40	50	<0.0001
	GP	41	–	–	–	–	–	–	48	–	–	40	–	
	GF	8	–	–	–	–	69	–	4	–	–	–	–	
	FR	5	–	19	–	–	–	14	4	–	50	–	–	
	BR	3	–	–	–	–	–	–	–	100	50	–	–	
	SR	–	6	–	–	–	31	–	7	–	–	–	–	
	Others/na	16	12	12	–	–	–	–	18	–	–	20	50	

F, female; M, male; Hetero., heterosexual; MSM, men who have sex with men; na, not available. MQ, Martinique; GP, Guadeloupe; GF, French Guiana; FR, mainland France; BR, Brazil; SR, Suriname.

## 4. Discussion

This study reveals that a large variety of HIV-1 non-B subtype strains, comprising eight subtypes, twelve CRFs, and various URFs have been introduced at multiple times in CSAFOT. Excepting Cuba ( Pérez, 2006 ) such diversity has not been observed previously in the Caribbean region. Prevalence of non-B subtypes has significantly increased over time in Martinique and Guadeloupe, reaching about 15–25% of HIV-infected subjects enrolled after 2010. This pattern resembles the growing complexity of the HIV-1 molecular epidemiologic scenario observed in mainland France, where prevalence of non-B subtypes has also increased over time (Chaix et al. 2013; Brand et al. 2014; Chaillon et al. 2017; Visseaux et al. 2020). Thus, despite the small size of the HIV epidemic in CSAFOT, this region exhibits a complex and changing HIV-1 molecular epidemiologic profile.

In CSAFOT, the most prevalent HIV-1 non-B clades were successfully spread by multiple active local and regional transmission chains and 12 major transmission networks drove most of CRF02_AG, A3, C, D, and F1 infections. Some of the major non-B subtype CSAFOT transmission clusters comprised sequences from a single territory, while others comprised sequences from two or three different CSAFOT. Our analyses support frequent viral exchanges between Martinique and Guadeloupe and more sporadic dissemination of non-B subtypes between the two French Caribbean islands and French Guiana, which is fully consistent with the overall population mobility in CSAFOT (Marie and Temporal 2011).

Phylogeographic analyses support frequent non-B subtype viral transmissions between CSAFOT and mainland France. Two CRF02 clusters most probably arose in mainland France and were independently disseminated to Martinique and Guadeloupe, while three clusters most probably arose in French Caribbean islands and were disseminated to mainland France. The most notable example was cluster CRF02_I_ for which we estimate a total of 13 possible transatlantic disseminations events. This cluster, comprising only men, most probably arose in Guadeloupe and from there it was disseminated to Martinique and mainland France multiple times. We also detected multiple viral exchanges of CRF02_I_ between Martinique and mainland France and at least one dissemination event from mainland France to French Guiana. Despite its recent origin (around the mid-2000s), cluster CRF02_I_ was able to disseminate and establish local transmissions in all French territories.

The CRF02_AG was the most prevalent and successfully disseminated HIV-1 non-B subtype in Martinique and Guadeloupe, accounting for 45% of non-B subtype infections in the region. Previous studies have demonstrated that CRF02_AG is the most prevalent non-B subtype in France, representing about 60% of non-B infections, and further showed that MSM individuals infected in France are involved in the local transmission of this non-B subtype (Brand et al. 2014, 2017; Chaillon et al. 2017). This study revealed that CRF02_AG is spreading among both MSM and heterosexual local transmission networks in Martinique and Guadeloupe. Three CRF02 clusters probably arose in Guadeloupe and mainland France and seem to be more actively spreading among MSM individuals. By contrast, two other clusters probably arose in Martinique and mainly disseminated among heterosexual individuals.

The subtype A3 established the largest non-B transmission cluster identified in French Guiana and its origin was traced to the late 1990s. Subtype A3 is a distinct subtype A clade quite frequent (5–30%) among HIV-infected subjects in several West African countries (Meloni et al. 2004). This A3 cluster identified in French Guiana represents the first recognized outbreak of subtype A3 outside West Africa. Notably, we detected five additional introductions of subtype A3 strains in French Guiana, some of them associated with individuals from West Africa (Benin, Guinea Bissau, and Ivory Coast), showing that dissemination of this viral lineage into French Guiana is not a rare phenomenon. Epidemiological data also support a dissemination of this subtype A3 lineage in the border region between French Guiana and Suriname.

West and Central African regions, which concentrate most of the French-speaking African countries, appear to be the most probable source of all CRF02_AG clades as well as of A3_I_, D_I_, F1_III_, and F1_IV_ clusters circulating in CSAFOT. Remarkably, the clade C_I_ mostly comprising heterosexual individuals > 50 years old, was probably introduced into Guadeloupe around the early 2000s from East Africa (Burindi), as the other two largest subtype C epidemics in the Americas (Brazilian and Cuban). The origin of the Brazilian subtype C epidemic was traced to Burundi around the middle 1970s (Delatorre et al. 2013) and that of the Cuban subtype C epidemic to Ethiopia around the middle 1990s (Delatorre and Bello 2013). The historical links of CSAFOT with Burundi are scarce and all individuals within the cluster C_I_ were of French origin.

All non-B subtype clusters circulating in French Caribbean islands are mostly or exclusively composed of individuals of French origin, with exception of the subtype D_I_ cluster that comprised a significant proportion (19%) of migrant individuals from Congo, Dominica, and Suriname that probably got infected after migration into the French Caribbean region. The subtype D_I_ transmission cluster was probably introduced in Guadeloupe from Central Africa around the late 1970s, constituting the oldest non-B subtype lineage detected in CSAFOT, consistent with the high frequency of URFs_BD also detected in that region. This transmission cluster comprises a significant fraction of both heterosexual (65%) and MSM (31%) individuals of different ages, supporting that this lineage is not restricted to a specific population group. Another peculiarity is that most (92%) of subtype D_I_-infected individuals were symptomatic, which may reflect longstanding chronic infections and/or faster disease progression rate (Amornkul et al. 2013).

The large number of Brazilian immigrants living in French Guiana may provide an epidemiological link for sporadic transmissions of non-B subtype strains from Brazil into French Guiana (Nacher et al. 2018). Consistent with this hypothesis, nearly all subtype F1 sequences and a great proportion of subtype C (29%) sequences detected in French Guiana were nested with Brazilian subtype F1 and subtype C clades. Clusters F1_I_ and F1_II_ detected in French Guiana mostly comprise sequences from Brazilian individuals living in French Guiana and in neighboring Northern Brazilian states. This supports that F1_I_ and F1_II_ were transmission clusters probably driven by Brazilian migrants that frequently travel across the French Guiana–Brazilian border, but these lineages seem to have a very restricted transmission to the native French Guianese population.

A previous study supports that competition of HIV-1 strains at the epidemic level may involve an advantage for the strain that was the first to colonize a population (Ferdinandy et al. 2015). The timing of introductions of different non-B subtype strains in CSAFOT that range from the late 1970s to the early 2000s, however, was clearly not related with the size of current non-B subtype epidemic outbreaks. The non-B subtype strain most successfully disseminated in the CSAFOT (CRF02_I_) started to spread much later than other non-B viral clades. The epidemic expansion of different clusters may have also been influenced by the virus genotype (Bertels et al. 2018). However, we failed to detect significant differences in viral load between different transmission clusters. These observations suggest that the characteristics of the existing contact transmission network are probably the most determinant driving force of the epidemic expansion of different HIV-1 non-B subtype lineages introduced in CSAFOT.

Although this study covers an important fraction of all HIV diagnosed individuals in Martinique (51%) and Guadeloupe (31%) over the study period, one important limitation is the variable sampling density along time which may introduce some bias in our results. Sampling density significantly increased between the mid-1990s and the middle 2000s in Martinique and between the mid-1990s and the late 2000s in Guadeloupe (Supplementary Fig. 4). The increasing prevalence of non-B variants in Martinique between 2006–2008 and 2015–2018, however, could be not explained by differences in sampling density as this remained roughly constant in that period. Another limitation of our study is the limited access to sequences from French Guiana of some specified non-B subtypes and sampled over a narrow time interval (2006–2012) that did not permit us to provide a picture of the overall prevalence and temporal trend of non-B subtypes in this French territory.

The results of this study offer important insights into HIV diversity trends in CSAFOT. Our data highlight that prevalence of HIV-1 non-B subtype variants has significantly increased over time in Martinique and Guadeloupe. The HIV-1 non-B subtype epidemic in CSAFOT resulted from independent introductions of a large variety of subtypes, CRFs, and URFs as well as from the subsequent local and regional expansion of some viral strains that were sequentially introduced between the late 1970s and the middle of the 2000s and spread through MSM and heterosexual contact networks. Our study emphasizes that early detection and treatment of people being part of the largest transmission chains combined with a coordinated regional healthcare response and pre-exposure prophylaxis in high-risk groups should have a significant impact on reducing the local spread of non-B subtypes that are continuously introduced in CSAFOT.

## Supplementary data

Supplementary data are available at *Virus Evolution* online.

## Supplementary Material

veaa081_Supplementary_Data
