# Supplementary material for: Increasing prevalence and local transmission of non-B HIV-1 subtypes in the French Antilles and French Guiana between 1995 and 2018
Source: Virus Evol. 2020 Nov 27;6(2):veaa081. doi: 10.1093/ve/veaa081 (PMC7724245; doi:10.1093/ve/veaa081)
Supplement: veaa081_Supplementary_Data [file veaa081_supplementary_data.docx]

**Supplementary Methods**

**HIV-1 subtyping.** In bootscanning analysis, the branch supports with HIV-1 reference sequences were determined through Neighbor-Joining trees based on 100 re-samplings, within a 250 bp window moving in steps of 10 bases. The ML trees were reconstructed using an online web server [1] under the best fit nucleotide substitution model selected with the SMS tool [2], the SPR branch-swapping algorithm of heuristic tree search, and the approximate likelihood-ratio test (a*LRT*) [3] of reliability tree topology. The ML trees were visualized using the FigTree v1.4.3 program [4].

**Spatiotemporal Reconstructions.** Because regression analyses using program TempEst v1.5.3 [5] revealed that most HIV-1 *pol* datasets here compiled do not contain sufficient temporal signal for reliable time-scale estimations (Figure S1), the time-scale was reconstructed using a uniform prior distribution on the substitution rate that encompasses mean values previously estimated for HIV-1 group M *pol* gene (2.0–3.0 × 10^-3^ subst./site/year) [6]. Bayesian MCMC analyses were performed using the GTR+I+Γ nucleotide substitution model [7], a Bayesian Skyline coalescent tree prior [8] and a relaxed uncorrelated lognormal molecular clock model [9]. Migration events throughout the phylogenetic histories were reconstructed using a reversible discrete phylogeography model [10] with a CTMC rate reference prior [11].

**References**

1. Guindon S, Lethiec F, Duroux P, Gascuel O. PHYML Online--a web server for fast maximum likelihood-based phylogenetic inference. Nucleic Acids Res. 2005 Jul 1;33(Web Server issue):W557-9.

2. Lefort V, Longueville JE, Gascuel O. SMS: Smart Model Selection in PhyML. Mol Biol Evol. 2017 Sep 1;34(9):2422-4.

3. Anisimova M, Gascuel O. Approximate likelihood-ratio test for branches: A fast, accurate, and powerful alternative. *Syst Biol* 2006,**55**:539-552.

4. Rambaut A. FigTree v1.4: Tree Figure Drawing Tool. *Available from* <http://tree.bio.ed.ac.uk/software/figtree/> 2009.

5. Rambaut A, Lam TT, Carvalho LM, Pybus OG. Exploring the temporal structure of heterochronous sequences using TempEst (formerly Path-O-Gen). Virus Evolution. 2016;2(1):vew007.

6. Hue S, Pillay D, Clewley JP, Pybus OG. Genetic analysis reveals the complex structure of HIV-1 transmission within defined risk groups. *Proc Natl Acad Sci U S A* 2005,**102**:4425-4429.

7. Tavaré S. Some probabilistic and statistical problems in the analysis of DNA sequences. p. . In: Miura RM, editor Some mathematical questions in biology—DNA sequence analysis Providence (RI): American Mathematical Society. 1986:p. 57–86.

8. Drummond AJ, Rambaut A, Shapiro B, Pybus OG. Bayesian coalescent inference of past population dynamics from molecular sequences. Mol Biol Evol. 2005 May;22(5):1185-92.

9. Drummond AJ, Ho SY, Phillips MJ, Rambaut A. Relaxed phylogenetics and dating with confidence. PLoS Biol. 2006 May;4(5):e88.

10. Lemey P, Rambaut A, Drummond AJ, Suchard MA. Bayesian phylogeography finds its roots. PLoS Comput Biol. 2009 Sep;5(9):e1000520.

11. Ferreira MAR, M.A. S. Bayesian analysis of elapsed times in continuous-time Markov chains. Canadian Journal of Statistics. 2008;26:355-68.

**Supplementary Data**

**Supplementary Figure 1.**

**Root-to-tip divergence of each HIV-1 clade dataset.** Correlations between the sampling date of each sequence and the genetic distance of that sequence from the root of the ML phylogeny obtained for each non-B subtype sequence dataset used for time-scale phylogeographic reconstructions. The determination coefficients (R^2^) is at the top left of each graph.

Ref.19_cpx.CU.99.CU7.AY894994

Ref.25_cpx.SA.03.J11451.EU6979

Ref.11_cpx.CM.96.96CM_4496.AF4

Ref.25_cpx.SA.03.J11233.EU6979

Ref.D.CD.83.ELI.K03454

Ref.D.CM.01.01CM_4412HAL.AY371

Ref.06_cpx.GH.03.03GH173_06.AB

Ref.37_cpx.CM.00.00CMNYU926.EF

Ref.J.SE.93.SE9280_7887.AF0823

Ref.C.BR.92.BR025_d.U52953

Ref.24_BG.ES.08.X2456_2.FJ6705

Ref.01_AE.CN.05.05GX001.GU5642

Ref.F1.FR.96.96FR_MP411.AJ2492

Ref.14_BG.PT.00.00PTHDE10.GU23

Ref.G.KE.93.HH8793_12_1.AF0616

Ref.K.CM.96.96CM_MP535.AJ24923

Ref.F2.CM.97.CM53657.AF377956

Ref.F1.BR.93.93BR020_1.AF00549

Ref.C.IN.95.95IN21068.AF067155

Ref.11_cpx.CM.95.95CM_1816.AF4

Ref.18_cpx.CU.99.CU68.AY894993

Ref.18_cpx.CU.99.CU14.AY586541

Ref.H.BE.93.VI991.AF190127

Ref.09_cpx.GH.96.96GH2911.AY09

Ref.02_AG.NG.x.IBNG.L39106

Ref.B.FR.83.HXB2_LAI_IIIB_BRU.

Ref.B.NL.00.671_00T36.AY423387

Ref.12_BF.AR.99.ARMA159.AF3859

Ref.14_BG.ES.00.X623.AF450097

Ref.B.US.98.1058_11.AY331295

Ref.B.TH.90.BK132.AY173951

Ref.A1.AU.03.PS1044_Day0.DQ676

Ref.H.CF.90.056.AF005496

Ref.C.ET.86.ETH2220.U46016

Ref.09_cpx.SN.95.95SN1795.AY09

Ref.02_AG.CM.99.pBD6_15.AY2716

Ref.13_cpx.CM.02.02CM_A1394.DQ

Ref.F1.BE.93.VI850.AF077336

Ref.A2.CM.01.01CM_1445MV.GU201

Ref.A1.UG.92.92UG037.AB253429

Ref.18_cpx.CM.97.CM53379.AF377

Ref.H.BE.93.VI997.AF190128

Ref.19_cpx.CU.99.CU29.AY588971

Ref.F1.FI.93.FIN9363.AF075703

Ref.09_cpx.CI.00.00IC_10092.AJ

Ref.11_cpx.CM.97.MP818.AJ29171

Ref.12_BF.AR.97.A32989.AF40863

Ref.A2.CD.97.97CDKTB48.AF28623

Ref.19_cpx.CU.99.CU38.AY588970

Ref.09_cpx.US.99.99DE4057.AY09

Ref.06_cpx.AU.96.BFP90.AF06469

Ref.D.UG.94.94UG114.U88824

Ref.02_AG.LR.x.POC44951.AB4856

Ref.H.GB.00.00GBAC4001.FJ71170

Ref.C.ZA.04.04ZASK146.AY772699

Ref.06_cpx.EE.01.EE0359.AY5356

Ref.01_AE.TH.90.CM240.U54771

Ref.G.NG.92.92NG083.U88826

Ref.24_BG.CU.03.CB471.AY900575

Ref.A1.RW.92.92RW008.AB253421

Ref.A2.CY.94.94CY017_41.AF2862

Ref.F2.CM.95.95CM_MP257.AJ2492

Ref.13_cpx.CM.96.96CM_1849.AF4

Ref.G.BE.96.DRCBL.AF084936

Ref.24_BG.CU.03.CB378.AY900574

Ref.12_BF.AR.97.A32879.AF40862

Ref.D.TZ.01.A280.AY253311

Ref.G.PT.x.PT2695.AY612637

Ref.F2.CM.95.95CM_MP255.AJ2492

Ref.J.CD.97.J_97DC_KTB147.EF61

Ref.J.CM.04.04CMU11421.GU23707

Ref.14_BG.ES.00.X605.AF450096

Ref.K.CD.97.97ZR_EQTB11.AJ2492

Ref.37_cpx.CM.97.CM53392.AF377

Ref.01_AE.AF.07.569M.GQ477441

Ref.13_cpx.CM.04.04CM_632_28.D

Ref.25_cpx.CM.06.06CM_BA_040.E

Ref.F2.CM.02.02CM_0016BBY.AY37

**Ma**

**r**

**tinique**

**Guadeloupe**

**Re**

**f**

**erences**

**0.93**

**CRF02_**

**A**

**G**

**1.00**

**0.99**

**0.95**

**0.87**

**CRF37_cpx**

**CRF25_cpx**

**CRF14_BG**

**Subtype G**

**CRF06_cpx**

**0.99**

**1.00**

**0.99**

**Subtype C**

**Subtype H**

**CRF11_cpx**

**CRF13_cpx**

**Subtype J**

**0.95**

**0.96**

**0.89**

**0.99**

**0.88**

**CRF09_cpx**

**CRF01_AE**

**Subtype K**

**Subsubtype A1**

**Subsubtype A2**

**CRF12_BF**

**Subsubtype F1**

**Subsubtype F2**

**CRF18_cpx**

**BD and BF recombinants**

**Subtype D**

**0.98**

**0.92**

**1.00**

**1.00**

**0.96**

**0.93**

**0.95**

**0.24**

**0.97**

**1.00**

**CRF19_cpx**

**CRF24_BG**

**1.00**

**0.03**

**Supplementary Figure 2. Subtype classification of the non-B sequences.** The maximum-likelihood tree was made with the non-B sequences from Guadeloupe and Martinique (yellow and red branches, respectively) and HIV-1 reference sequences of pure subtypes and CRFs (black branches). Each HIV-1 clade is highlighted with a gray square with its *aLRT* branch support value indicated. Horizontal branch lengths are drawn to scale with the bar at the bottom indicating nucleotide substitutions per site.

**Supplementary Table 1. HIV-1 *pol* subtypes in Martinique and Guadeloupe.**

| **HIV-1 clade** | | **Martinique**  **(N = 1,432)** | **Guadeloupe**  **(N = 1,025)** |
| --- | --- | --- | --- |
| Subtypes | B | 1,247 (87.1%) | 895 (87.3%) |
|  | A1 | 3 (0.2%) | 1 (0.1%) |
|  | A2 | 2 (0.1%) | - |
|  | A3 | 2 (0.1%) | 2 (0.1%) |
|  | C | 11 (0.8%) | 10 (1.0%) |
|  | D | 13 (0.9%) | 14 (1.4%) |
|  | F1 | 8 (0.6%) | 2 (0.1%) |
|  | G | 4 (0.3%) | 4 (0.4%) |
|  | H | 1 (0.1%) | - |
|  | Total | 1,291 (90.3%) | 928 (90.5%) |
| CRFs | CRF02_AG | 97 (6.8%) | 45 (4.4%) |
|  | CRF01_AE | 6 (0.4%) | 2 (0.1%) |
|  | CRF06_cpx | 1 (0.1%) | - |
|  | CRF09_cpx | - | 1 (0.1%) |
|  | CRF11_cpx | 3 (0.2%) | - |
|  | CRF12_BF | 1 (0.1%) | - |
|  | CRF13_cpx | - | 1 (0.1%) |
|  | CRF18_cpx | 1 (0.1%) | - |
|  | CRF19_cpx | - | 4 (0.4%) |
|  | CRF24_cpx | 1 (0.1%) | - |
|  | CRF25_cpx | - | 3 (0.3%) |
|  | CRF37_cpx | 3 (0.2%) | 1 (0.1%) |
|  | Total | 113 (7.9%) | 57 (5.6%) |
| URFs | URF_AG | 3 (0.2%) | 4 (0.4%) |
|  | URF_BA | 3 (0.2%) | - |
|  | URF_BC | 2 (0.1%) | - |
|  | URF_BD | 3 (0.2%) | 16 (1.6%) |
|  | URF_BF | 14 (1.0%) | 16 (1.6%) |
|  | URF_BG | 1 (0.1%) | 2 (0.1%) |
|  | URF_FD | 1 (0.1%) | - |
|  | URF_FG | 1 (0.1%) | 1 (0.1%) |
|  | URF_JK | - | 1 (0.1%) |
|  | Total | 28 (2.0%) | 40 (3.9%) |

**Supplementary Table 2. Prevalence of HIV-1 subtype B and non-B *pol* sequences across different time-intervals in Martinique and Guadeloupe.**

| **Region** | **Time period** | **Subtype B** | **Non-B Subtypes** |
| --- | --- | --- | --- |
| Martinique | 1995-2002  (N = 251) | 236  (94%) | 15  (6%) |
|  | 2003-2005  (N = 257) | 237  (92%) | 20  (8%) |
|  | 2006-2008  (N = 257) | 232  (90%) | 25  (10%) |
|  | 2009-2011  (N = 215) | 185  (86%) | 30  (14%) |
|  | 2012-2014  (N = 223) | 184  (83%) | 39  (17%) |
|  | 2015-2018  (N = 229) | 173  (76%) | 56  (24%) |
|  | 1995-2018  (N = 1,432) | 1,247  (87%) | 185  (13%) |
| Guadeloupe | 1999-2005  (N = 237) | 218  (92%) | 19  (8%) |
|  | 2006-2008  (N = 258) | 228  (88%) | 30  (12%) |
|  | 2009-2011  (N = 298) | 257  (86%) | 41  (14%) |
|  | 2012-2014  (N = 232) | 192  (83%) | 40  (17%) |
|  | 1999-2014  (N = 1,025) | 895  (87%) | 130  (13%) |

**Supplementary Figure 3A :** Maximum likelihood (ML) phylogenetic trees for CRF02_AG sequences recovered from CSAFOT and other key geographic regions. The colors of the terminal branches represent the geographic origin of each sequence as indicated in the legend of each tree. The local/regional main clusters are indicated by shaded boxes with their a*LRT* branch support values. The branch lengths are drawn to scale with the bar in the center indicating nucleotide substitutions per site.


**Supplementary Figure 3B:** Maximum likelihood (ML) phylogenetic trees for A sequences recovered from CSAFOT and other key geographic regions. The colors of the terminal branches represent the geographic origin of each sequence as indicated in the legend of each tree. The local/regional main clusters are indicated by shaded boxes with their a*LRT* branch support values. The branch lengths are drawn to scale with the bar in the center indicating nucleotide substitutions per site.

**Supplementary Figure 3C:** Maximum likelihood (ML) phylogenetic trees for C sequences recovered from CSAFOT and other key geographic regions. The colors of the terminal branches represent the geographic origin of each sequence as indicated in the legend of each tree. The local/regional main clusters are indicated by shaded boxes with their a*LRT* branch support values. The branch lengths are drawn to scale with the bar in the center indicating nucleotide substitutions per site.

**Supplementary Figure 3D:** Maximum likelihood (ML) phylogenetic trees for D sequences recovered from CSAFOT and other key geographic regions. The colors of the terminal branches represent the geographic origin of each sequence as indicated in the legend of each tree. The local/regional main clusters are indicated by shaded boxes with their a*LRT* branch support values. The branch lengths are drawn to scale with the bar in the center indicating nucleotide substitutions per site.
**Supplementary Figure 3E:** Maximum likelihood (ML) phylogenetic trees for F1 sequences recovered from CSAFOT and other key geographic regions. The colors of the terminal branches represent the geographic origin of each sequence as indicated in the legend of each tree. The local/regional main clusters are indicated by shaded boxes with their a*LRT* branch support values. The branch lengths are drawn to scale with the bar in the center indicating nucleotide substitutions per site.

**Supplementary Table 3. HIV-1 CRF02_AG, subtype A, subtype C, subtype D and subtype F1 *pol* sequences used in ML phylogenetic analyses.**

| **Clade** | **Americas** | **n** | **Africa/Asia** | **n** | **Europe** | **n** |
| --- | --- | --- | --- | --- | --- | --- |
| CRF02_AG | Brazil | 29 | Benin | 92 | France | 769 |
|  | Colombia | 2 | Burkina Faso | 132 |  |  |
|  | Cuba | 3 | Cameroon | 827 |  |  |
|  | Ecuador | 9 | Côte d'Ivoire | 55 |  |  |
|  | French Guiana | 27 | CD^1^ | 17 |  |  |
|  | Guadeloupe | 45 | Equatorial Guinea | 21 |  |  |
|  | Martinique | 97 | Gabon | 58 |  |  |
|  | Mexico | 1 | Ghana | 184 |  |  |
|  |  |  | Liberia | 48 |  |  |
|  |  |  | Mali | 160 |  |  |
|  |  |  | Mauritania | 35 |  |  |
|  |  |  | Nigeria | 173 |  |  |
|  |  |  | Senegal | 272 |  |  |
|  |  |  | Togo | 116 |  |  |
|  |  |  | Others^2^ | 14 |  |  |
| A | Cuba | 4 | Benin | 4 | France | 5 |
|  | French Guiana | 21 | Burkina Faso | 7 |  |  |
|  | Guadeloupe | 3 | Burundi | 25 |  |  |
|  | Martinique | 7 | Cameroon | 112 |  |  |
|  |  |  | Central African Republic | 18 |  |  |
|  |  |  | CD^1^ | 55 |  |  |
|  |  |  | Gabon | 12 |  |  |
|  |  |  | Ghana | 13 |  |  |
|  |  |  | Kenya | 656 |  |  |
|  |  |  | Mali | 11 |  |  |
|  |  |  | Rwanda | 172 |  |  |
|  |  |  | Senegal | 26 |  |  |
|  |  |  | Tanzania | 230 |  |  |
|  |  |  | Togo | 22 |  |  |
|  |  |  | Uganda | 2,124 |  |  |
|  |  |  | Others^3^ | 18 |  |  |
| C | Brazil | 102 | Burundi | 92 | France | 6 |
|  | Cuba | 34 | CD^1^ | 22 |  |  |
|  | French Guiana | 7 | India | 65 |  |  |
|  | Guadeloupe | 10 | Zambia | 146 |  |  |
|  | Martinique | 11 |  |  |  |  |
| D | Brazil | 18 | Cameroon | 61 |  |  |
|  | French Guiana | 4 | CD^1^ | 28 |  |  |
|  | Guadeloupe | 14 | Kenya | 126 |  |  |
|  | Martinique | 13 | Tanzania | 160 |  |  |
|  |  |  | Uganda | 1,545 |  |  |
|  |  |  | Others^4^ | 15 |  |  |
| F1 | Brazil | 338 | Angola | 25 | France | 6 |
|  | Cuba | 5 | Others^5^ | 14 | Romania | 332 |
|  | French Guiana | 28 |  |  |  |  |
|  | Guadeloupe | 2 |  |  |  |  |
|  | Martinique | 8 |  |  |  |  |

^1^ CD stands for Democratic Republic of the Congo, following the ISO 3166 international standard.

^2^ CRF02_AG: Angola, Chad, Gambia, Guinea and Guinea Bissau.

^3^ Subtype A: Chad, Equatorial Guinea, Nigeria, Sudan and Tunisia.

^4^ Subtype D: Burundi, Central Africa, Gabon and Rwanda.

^5^ Subtype F1: Cameroon, CD, Central Africa, Republic of Congo, Gabon, Nigeria and Senegal.

**Supplementary Table 4. Phylogenetic clustering of HIV-1 CRF02_AG, subtype A, subtype C, subtype D and subtype F1 pol sequences from French Antilles, French Guiana and mainland France.**

| **Clade** | **Cluster** | **MQ^1^** | **GP** | **GF** | **FR** | **Total** |
| --- | --- | --- | --- | --- | --- | --- |
| CRF02_AG | CRF02_I_ | 15 (15%) | 20 (44%) | 6 (22%) | 30 (4%) | 71 (8%) |
|  | CRF02_II_ | 19 (20%) | 1 (2%) | - | 14 (2%) | 34 (4%) |
|  | CRF02_III_ | 16 (16%) | - | - | - | 16 (2%) |
|  | CRF02_IV_ | 5 (5%) | 1 (2%) | - | 7 (1%) | 13 (1%) |
|  | CRF02_V_ | 6 (6%) | 2 (4%) | - | - | 8 (1%) |
|  | Others (n ≥ 5)^2^ | 6 (6%) | 2 (4%) | - | 265 (34%) | 273 (29%) |
|  | Small (n < 5) | 11 (11%) | 8 (18%) | 6 (22%) | 98 (13%) | 123 (13%) |
|  | Non-clustered | 19 (20%) | 11 (24%) | 15 (56%) | 355 (46%) | 400 (43%) |
|  | Total | 97 (100%) | 45 (100%) | 27 (100%) | 769 (100%) | 938 (100%) |
| A | A3_I_ | - | - | 13 (65%) | - | 13 (37%) |
|  | Small (n < 5) | 3 (43%) | 1 (33%) | 2 (10%) | 1 (20%) | 7 (20%) |
|  | Non-clustered | 4 (57%) | 2 (67%) | 5 (25%) | 4 (80%) | 15 (43%) |
|  | Total | 7 (100%) | 3 (100%) | 20 (100%) | 5 (100%) | 35 (100%) |
| C | C_I_ | 7 (64%) | 9 (90%) | - | - | 16 (48%) |
|  | Small (n < 5) | - | - | 2 (33%) | - | 2 (6%) |
|  | Non-clustered | 4 (36%) | 1 (10%) | 4 (67%) | 6 (100%) | 15 (46%) |
|  | Total | 11 (100%) | 10 (100%) | 6 (100%) | 6 (100%) | 33 (100%) |
| D | D_I_ | 10 (77%) | 14 (100%) | 3 (75%) | - | 27 (87%) |
|  | Small (n < 5) | 2 (15%) | - | - | - | 2 (6%) |
|  | Non-clustered | 1 (8%) | - | 1 (25%) | - | 2 (6%) |
|  | Total | 13 (100%) | 14 (100%) | 4 (100%) | - | 31 (100%) |
| F1 | F1_I_ | - | - | 9 (32%) | - | 9 (20%) |
|  | F1_II_ | - | - | 7 (25%) | - | 7 (16%) |
|  | F1_III_ | 3 (38%) | 2 (100%) | 1 (4%) | 1 (17%) | 7 (16%) |
|  | F1_IV_ | 4 (50%) | - | - | 1 (17%) | 5 (11%) |
|  | Small (n < 5) | - | - | 4 (14%) | - | 4 (9%) |
|  | Non-clustered | 1 (12%) | - | 7 (25%) | 4 (67%) | 12 (27%) |
|  | Total | 8 (100%) | 2 (100%) | 28 (100%) | 6 (100%) | 44 (100%) |

^1^MQ stands for Martinique, GP for Guadeloupe, GF for French Guiana and FR for France, following the ISO 3166 international standard.

^2^CRF02_AG French clusters of medium/large size that comprise a very low proportion (< 5%) of sequences from CSAFOT or that only contain sequences from mainland France.

**Supplementary Table 5. Origins of HIV-1 CRF02_AG, subtype A, subtype C, subtype D and subtype F1 *pol* sequences used in Bayesian phylogeographic analyses.**

| **Clade** | **Americas** | ***N*** | **Africa** | ***N*** | **Europe** | ***N*** |
| --- | --- | --- | --- | --- | --- | --- |
| CRF02_AG | French Guiana | 6 | Burkina Faso | 1 | France | 68 |
|  | Guadeloupe | 24 | Benin | 2 |  |  |
|  | Martinique | 61 | Ivory Coast | 4 |  |  |
|  |  |  | Cameroon | 8 |  |  |
|  |  |  | Gabon | 1 |  |  |
|  |  |  | Ghana | 5 |  |  |
|  |  |  | Liberia | 4 |  |  |
|  |  |  | Mali | 13 |  |  |
|  |  |  | Mauritania | 1 |  |  |
|  |  |  | Nigeria | 7 |  |  |
|  |  |  | Chad | 1 |  |  |
|  |  |  | Togo | 4 |  |  |
| A | French Guiana | 13 | Benin | 3 |  |  |
|  |  |  | Burkina Faso | 7 |  |  |
|  |  |  | Cameroon | 4 |  |  |
|  |  |  | Chad | 1 |  |  |
|  |  |  | Equatorial Guinea | 3 |  |  |
|  |  |  | Ghana | 13 |  |  |
|  |  |  | Mali | 11 |  |  |
|  |  |  | Nigeria | 5 |  |  |
|  |  |  | Senegal | 5 |  |  |
|  |  |  | Togo | 21 |  |  |
| C | Guadeloupe | 9 | Burundi | 92 |  |  |
|  | Martinique | 7 | Ethiopia | 47 |  |  |
|  |  |  | Kenya | 33 |  |  |
|  |  |  | Tanzania | 43 |  |  |
|  |  |  | Uganda | 37 |  |  |
| D | Brazil | 15 | Cameroon | 58 |  |  |
|  | French Guiana | 4 | Central African Republic | 1 |  |  |
|  | Guadeloupe | 14 | CD^1^ | 21 |  |  |
|  | Martinique | 12 | Gabon | 3 |  |  |
|  |  |  | Kenya | 6 |  |  |
|  |  |  | Tanzania | 2 |  |  |
|  |  |  | Uganda | 2 |  |  |
| F1 | Brazil | 208 | Angola | 25 | France | 2 |
|  | French Guiana | 17 | Cameroon | 3 | Romania | 25 |
|  | Guadeloupe | 2 | Central African Republic | 2 |  |  |
|  | Martinique | 7 | Congo | 1 |  |  |
|  |  |  | CD^1^ | 4 |  |  |

^1^ CD stands for Democratic Republic of the Congo, following the ISO 3166 international standard.

**Supplementary Table 6** Clades’ branch support values in ML and Bayesian analysis.

| **Subtype/CRF** | **Clade** | **aLRT support (ML)** | **Posterior probability (Bayesian)** |
| --- | --- | --- | --- |
| CRF02_AG | CRF02_I_ | 1.00 | 1.00 |
|  | CRF02_II_ | 0.96 | 1.00 |
|  | CRF02_III_ | 0.96 | 1.00 |
|  | CRF02_IV_ | 0.97 | 1.00 |
|  | CRF02_V_ | 0.99 | 1.00 |
| A | A3_I_ | 1.00 | 1.00 |
| C | C_I_ | 0.91 | 1.00 |
| D | D_I_ | 0.92 | 1.00 |
| F1 | F1_I_ | 0.99 | 0.98 |
|  | F1_II_ | 0.85 | 0.94 |
|  | F1_III_ | 0.97 | 1.00 |
|  | F1_IV_ | 0.89 | 0.99 |

**Supplementary Figure 4.
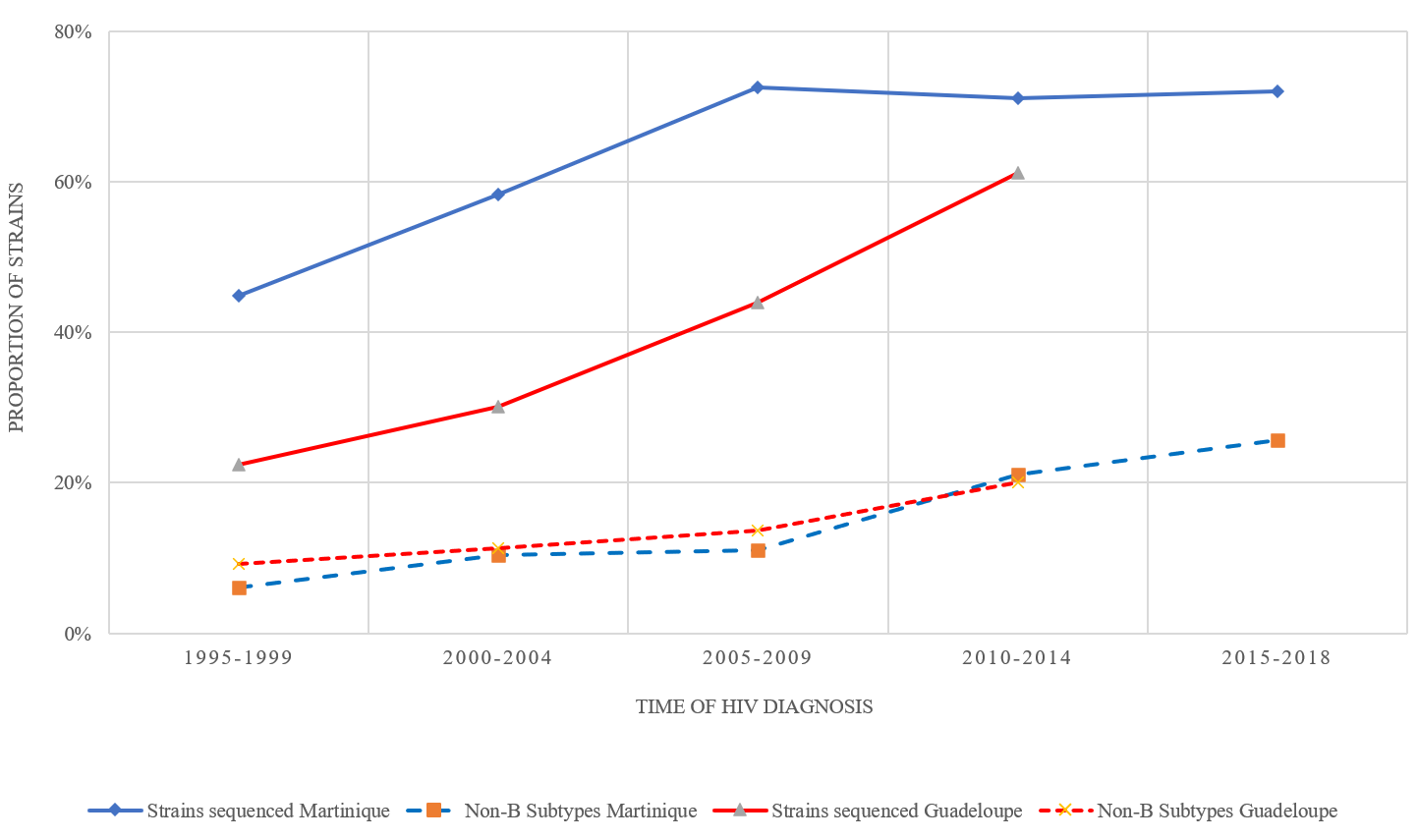
**

Percentage of strains sequenced and percentage of non-B strains over time in Guadeloupe and Martinique. Proportion are based on HIV diagnosis date of individuals. The linear Chi2 for trend showed a very significant increase of non-B strains over time (P<0.0001) for Martinique and for Guadeloupe.
